# Associations of Child Temperament with Child Overweight and Breakfast Habits: A Population Study in Five-Year-Olds

**DOI:** 10.3390/nu7125522

**Published:** 2015-12-03

**Authors:** Thea Steen Skogheim, Margarete Erika Vollrath

**Affiliations:** 1 Norwegian Institute of Public Health, Oslo 0403, Norway; theaskogheim@gmail.com; 2 Institute of Psychology, University of Oslo, Oslo 0373, Norway

**Keywords:** children, temperament, overweight, obesity, breakfast, cross-sectional

## Abstract

This study examines the associations of child temperament with overweight/obesity and breakfast habits. Participants were 17,409 five-year-olds whose mothers partake in the Norwegian Mother and Child Cohort Study (MoBa), and completed a questionnaire at the child’s 5th birthday. Temperament was assessed as externalizing, internalizing and sociable temperament. Breakfast habits differentiated between “every day”, “4 to 6 times a week”, and “0 to 3 times a week”. The child’s weight status was determined by Body Mass Index-percentiles and categorized as normal weight *versus* overweight/obese. Children with externalizing temperament had higher odds of being overweight and higher odds of not eating breakfast daily. Children high in internalizing temperament had higher odds of not eating breakfast daily, but not of being overweight. Children with average scores of sociability were more prone to being overweight but had normal breakfast habits. All results were adjusted for key confounders. That five-year-olds high in externalizing temperament had a higher risk to be overweight adds important information to the literature. The association of externalizing temperament with child breakfast habits so early in life is intriguing, as parents mostly control eating patterns in children that young. Mechanisms mediating this association should be explored.

## 1. Introduction

Childhood overweight and obesity are among the greatest global public health challenges of the 21st century [1]. In 2013, over 42 million children under the age of five were classified as overweight or obese [1]. Overweight results from a long-lasting excess of caloric intake in relation to caloric output, and is described as abnormal fat accumulation [1,2]. In children, overweight and obesity have usually been defined according to Body Mass Index (BMI), where BMI percentiles divide between underweight, normal weight, overweight and obese [1]. Children who are above the 85th and 95th percentiles of their normative BMI values with regard to sex and age, are classified as overweight and obese, respectively [3]. Children who are overweight or obese are at risk of mental and somatic health problems including asthma, diabetes, depression and being stigmatized and teased [4,5,6]. Furthermore, obesity in childhood increases the risk for continued obesity in adulthood [7].

Among the nutritional risk factors for childhood overweight are breakfast skipping, low consumption of fruit and vegetables, and increased consumption of confectionary, soft drinks, fruit juices and fast food [8]. Eating breakfast provides important nutrients to the child at the beginning of the day, which is needed for basic functioning and cognitive performance at school [9,10,11,12,13,14]. Moreover, not eating breakfast can lead to hunger pangs and make the children prone to overeating at the subsequent meal [15,16,17]. Predictors of children’s breakfast consumption have been examined infrequently. However, the parents’ education level/socioeconomic status has been identified as a predictor of breakfast skipping in children and adolescents [13,18,19].

Many risk factors for child overweight, for irregular eating patterns, and for the consumption of obesogenic foods, have been identified, most importantly environmental conditions such as the parents’ education, income and the child’s neighborhood conditions [13,20,21]. However, although parents are in control of child feeding, even younger children have strong means to refuse parent-chosen food and obtain the foods they prefer instead. In this struggle, the child’s characteristics in terms of temperament or behavior problems play a role [22]. Temperament is a biologically based and stable pattern of self-regulation and reactivity, which comprises emotional, attentional and motoric responses [23,24]. Temperament is present at birth but develops and differentiates as the child becomes older and is also affected and shaped by life experiences. Typical dimensions of temperament include “externalized negative emotionality” (e.g., aggressive and disobedient), “internalized negative emotionality” (e.g., fearful and sad), and “positive emotionality” (e.g., social and active), which is also called “surgency” or “extraversion” [23,25]. Conceptually, there is a strong overlap between behavioral problems and temperament because the two research traditions have developed in parallel but are broadly assessing the same traits [26,27,28].

Several studies have explored whether there is an association between overweight and temperament in infants and in children from one year of age up to 10 years of age. Some studies show positive associations between child temperament and weight [29,30,31,32,33,34,35,36,37,38,39,40,41,42,43] and some studies show no associations [44,45,46,47,48,49,50,51]. In spite of divergent findings, traits from the externalizing spectrum (negative emotions, impulsiveness, low self-regulation) are the most frequently associated with child overweight [29,31,34,35,36,37,40,42,43]. Surgency/extraversion has also been associated with greater weight gain and overweight [33,36,38]. Furthermore, internalizing temperament has been associated with both higher and lower likelihood [32] of being overweight [35].

Although the parents are in control of feeding their children, their choices of foods, food quantity, and timing of feeding is influenced by the child’s temperament, which in turn is associated with the child’s appetite, food preferences and cravings from early on. Studies found that children with impulsive, emotional, acting-out temperamental traits (externalizing temperament) were breastfed for a shorter period, were more likely to receive complementary foods early, consumed more carbohydrates, received sweet drinks at night more often, and received more sweets and sweet drinks during the day [41,42,48,52,53,54]. In our previous studies in 18- and 36-month-olds in the Norwegian Mother and Child Cohort Study (MoBa), we found that also internalizing temperament was associated with a higher likelihood of receiving or consuming sweet foods and sweet drinks [52,53]. These findings suggest a pattern of feeding by which the mothers intend to not only to provide for the child’s nutritional needs, but also to soothe the child’s negative emotions [55]. Whether child temperament is also related to evolving dietary habits such as eating breakfast has not been studied up to now.

Investigating temperament as a predictor for child overweight and eating breakfast around age five to six years is interesting for several reasons. In smaller children, obesity may need time to emerge, which may be the reason why several studies investigating infants and toddlers up to age three have not yielded significant associations between temperament and weight [45,47,48,50]. Moreover, during the period between five and seven years, children’s BMI undergoes the adiposity rebound, a period of falling BMI followed by a steep increase [56]. The steepness of this rebound is indicative for adult obesity [57].

In the present study, our first goal is to extend previous findings by examining the associations of child temperament with child overweight in a large sample of five-year-olds. The second goal is to examine the associations of child temperament with child breakfast habits.

## 2. Method Section

### 2.1. Study Design and Participants

The main data were derived from a large Norwegian pregnancy cohort study, which followed pregnant women and their children to the child’s fifth birthday. There are more than 100,000 children in the study, of which approximately 48,000 are expected to be retained when the five-year follow up data collection will be completed in 2016. In this study there were 18,047 participants, reduced to 17,409 after excluding multiple births and missing values on central variables. The study is known as the Norwegian Mother and Child Cohort Study (MoBa) conducted by the Norwegian Institute of Public Health, recruiting pregnant women from 1999 to 2008 [58]. Women were invited to participate when scheduling their first free ultrasound scanning at the 17th week of pregnancy. The participation rate among invited women was 42.7% [59]. There were three questionnaires during pregnancy and four questionnaires after birth. The questionnaires contained queries regarding the child, the mother, and also the father of the child. The study was approved by the Regional Committees for Medical and Health Research Ethics (REC/REK) in South-Eastern Norway. In this article, the core information bases on the 5-year-questionnaire (quality assured release #7 in 2014). Information from the other questionnaires was used for the imputation of missing values.

### 2.2. Outcomes

In order to create child BMI percentile scores, we used the growth curves for Norwegian children [60] to determine the 85th and 95th percentile overweight and obesity cut-off-points. Because we had few obese children for multivariate analyses (*n* = 171), we combined overweight and obese into one category. The underweight children were included in the normal weight category.

Breakfast consumption was measured with the query: “how often does the child eat breakfast (at home or in the kindergarten)?” The response categories were: “rarely/never”, “once a week”, “2–3 times a week”, “4–6 times a week”, and “every day”. We distinguished between three groups of breakfast eating: “every day”, “4 to 6 times a week”, and “0 to 3 times a week”.

### 2.3. Exposures

In parallel with our previous publications on temperament and eating in 18- and 36-month-olds from the MoBa study [52,53], we created measures of temperament by pooling 12 items from the Emotionality, Activity, and Sociability Questionnaire (EAS) and 24 items from the Child Behavior Checklist (CBCL). There are several advantages to this approach. Firstly, the EAS provides the dimensions of activity and sociability, which are lacking in the CBCL, but are important predictors of normal weight in children [41]. Secondly, the pooled scales are longer and hence more reliable. Thirdly the pooled scales will enable longitudinal comparisons in future studies on this material. The items of the CBCL have 3 response categories ranging between “does not apply at all” (0) and “applies a lot” (2); the items of the EAS have 5 response categories, ranging between “not typical” (0) and “very typical” (2), coded in 0.5 units to make the measures commensurate.

Temperament dimensions were created by determining the underlying structure of the items by principal component analysis (PCA) with Varimax rotation, then assembling the items with the highest loadings to scales and examining their reliability. The initial correlation matrix showed correlations above *r* = 0.30, the Kaiser-Meyer-Olkin (KMO) measure of sampling adequacy had a value of 0.86, and Bartlett’s test of sphericity was significant (*p* ≤ 0.01), indicating that the PCA produced reliable factors. Nine factors had an eigenvalue >1, but the Scree test indicated that 3 to 5 factors were optimal. A series of PCAs extracting 3 to 5 components showed that the loading pattern of the 3-factor solution was the most consistent with Rothbart’s theory [23] and with our previous findings [52,53]. The factors were externalizing temperament, internalizing temperament, and sociable temperament (sociability) (Table 1).

**Table 1:** Three factor solution of temperament: factor loadings for principal component analysis (PCA) with varimax rotation.

Item	Pattern Coefficients		
	Externalizing	Internalizing	Sociability
Can not sit still, restless or hyperactive	**0.68**	−0.04	−0.02
Can not concentrate, can’t pay attention for long	**0.61**	0.06	−0.06
Can not stand waiting; wants everything now	**0.60**	0.25	0.01
Quickly shifts from one activity to another	**0.57**	0.08	0.04
Demands must be met immediately	**0.54**	0.32	0.01
Your child is always on the go	**0.54**	−0.28	0.26
Your child is off and running as soon as he/she wakes up in the morning	**0.49**	−0.25	0.17
Gets in many fights	**0.47**	0.25	0.04
Punishment doesn’t change his/her behavior	**0.47**	0.14	−0.07
Your child reacts intensely when upset	**0.45**	0.44	0.02
Defiant	**0.45**	0.36	0.02
Hits others	**0.44**	0.10	−0.07
Your child prefers quiet, inactive games to more active ones	**−0.41**	0.32	−0.21
Gets into everything	**0.38**	0.22	0.19
Your child cries easily	0.07	**0.70**	0.00
Your child gets upset (or sad) easily	0.34	**0.65**	0.04
Cries a lot	0.12	**0.61**	0.06
Feelings are easily hurt	0.08	**0.58**	−0.02
Clings to adults or too dependent	0.21	**0.46**	−0.18
Too fearful or anxious	0.02	**0.45**	−0.17
Nervous, high strung, or tense	0.11	**0.44**	−0.12
Self-conscious or easily embarrassed	−0.02	**0.44**	−0.33
Afraid to try new things	−0.05	**0.43**	−0.29
Your child likes to be with people	−0.05	−0.03	**0.77**
Your child is very sociable	0.04	−0.17	**0.73**
Your child finds other people more stimulating than anything else	0.03	0.04	**0.71**
Your child prefer playing with others rather than alone	0.06	0.06	**0.60**
Your child is very friendly with strangers	0.02	−0.19	**0.60**
Your child takes a long time to warm up to strangers	−0.04	0.30	**−0.57**
Explained variance	17.2%	13.22%	6.46%

Note: Kaiser Meyer Olkin = 0.84. Bartlett’s test of sphericity <0.01; Total explained variance = 36.8%. The highest loadings on each factor are boldfaced.

After performing reliability analyses, four items were excluded and one item was assigned to another scale. Three items from the externalizing scale were excluded (“Your child is always on the go”, “Your child is off and running as soon as he/she wakes up in the morning”, “Your child prefers quiet, inactive games to more active ones”), the item “Your child takes a long time to warm up to strangers” was moved from the sociability scale to the internalizing scale, and “Your child is very friendly with strangers” was excluded from the sociability scale. The total amount of items forming the scales was thus reduced from 36 to 25.

The scales resulting from summarizing the items with the highest loadings on one factor had robust Cronbach’s alphas, with α = 0.80 for the externalizing scale (11 items), α = 0.75 for the internalizing scale (10 items), and α = 0.74 for the sociability scale (4 items). To achieve linearity of the log odds, the temperament scales were grouped into three categories, using interquartile range, with the first category including values below the 25th percentile, the second values between the 25th and the 75th percentile, and the last one with values above the 75th percentile.

### 2.4. Covariates

Covariates in the study included child gender, number of children in the household, maternal BMI, the education of both mother and father (reported by the mother), the mothers’ marital status during pregnancy, and maternal distress when the child was five years old (Table 2). The number of children in the household was retrieved from the 5-year questionnaire and categorized as 1 child, 2 children or 3 or more children. To determine the mothers’ weight at child age 5 years, we used the BMI classification by the World Health Organization [1] with underweight at BMI values below 18.5, normal weight at values from 18.5 to 24.9, overweight at values from 25 up to 30, and obese at values from 30 upwards [1]. There were rather few obese mothers (8.7%), therefore overweight and obesity was combined into one category. There were also few underweight mothers (2.8%), so they were included in the normal weight category. Parental education was retrieved from the questionnaire the mothers completed at the 17th week of pregnancy, where she reported both her own education and the education of the father of the child. Education was assessed as an ordinal variable with 7 response categories ranging from the obligatory 9 years of primary school to completed university/college education corresponding to 17 years of education. To combine the parents’ education, high and low education (less than college/university) was assigned to three categories: “low education in both parents”, “high education in one parent” (college/university), and “high education in both parents”. Information about the mothers’ marital status at child age 5 was dichotomized into “married/cohabiting” and “single/divorced/separated/widowed/other”. Mothers’ anxiety and depression at child age 5 was measured by means of the Hopkins Symptom Checklist-8 (SCL-8) [61].

**Table 2:** Characteristics of mothers and children regarding demographics, weight and breakfast habits.

	*n*	%	Mean	SD
**Child Variables**				
**BMI**				
Normal weight/underweight	15,197	87.3	15.26	1.04
Overweight	2041	11.7	17.81	0.51
Obese	171	1.0	19.97	0.79
**Breakfast (at home/kindergarten)**				
Every day	16,624	95.5		
Four to six times a week	545	3.1		
Two to three times a week	206	1.2		
Once a week	15	0.1		
Never/rarely	19	0.1		
**Internalizing temperament**				
Above 75th percentile	4511	25.9		
Between 25th and 75th percentile	8367	48.1		
Below 25th percentile	4531	26.0		
**Externalizing temperament**				
Above 75th percentile	4560	26.2		
Between 25th and 75th percentile	7708	44.3		
Below 25th percentile	5141	29.5		
**Sociable temperament**				
Above 75th percentile	6321	36.3		
Below 25th and 75th percentile	7429	42.7		
Under 25th percentile	3659	21.0		
**Number of children in household**				
One child	5872	33.7		
Two children	7337	42.1		
Three children or more	4200	24.1		
**Mother Variables**				
**Maternal BMI**				
Normal weight	12,062	69.3	21.82	1.82
Overweight	3833	22.0	26.98	1.38
Obese	1514	8.7	33.64	3.39
**Parental education (completed and ongoing education)**				
No parents higher education ^a^	4253	24.4		
One parent higher education	4997	28.7		
Both parents higher education	8159	46.9		
**Parental marital status**				
Married/partner (cohabitant)	16,951	97.4		
Divorced/separated/widow/single/other	458	2.6		
**Maternal SCL-8**	17,409		1.20	0.33

^a^ Higher education (college or university).

### 2.5. Statistical Analysis

All analyses were performed using SPSS version 22 (SPSS, Inc., Chicago, IL, USA). Missing values were imputed with the Expectation-Maximization (EM) algorithm. For child weight and height, missing values were imputed based on height and weight information available at birth and at child ages 6 months, 18 months, and 36 months. For maternal weight and height, missing height and weight data were imputed from her reports in the questionnaires at 17th week of pregnancy, and at child age 6 through 36 months. For mothers’ and fathers’ education, missing values were imputed based on income and civil status information from the 17th week questionnaire. Missing values were not imputed for child breakfast, child gender, number of children in the household, maternal marital status, and maternal SCL-8. All missing data were excluded from the analyses.

Binary logistic regression and multinomial logistic regression analyses were employed to compute the associations of temperament with child overweight and breakfast frequency. Regression analyses both adjusting and not adjusting for covariates were performed.

## 3. Results

### 3.1. Descriptive Statistics

The vast majority of the children (95.5%) ate breakfast every day, 3.1% ate breakfast 4–6 times a week, 1.2% ate breakfast 2–3 times a week, 0.1% ate breakfast once a week, and 0.1% ate breakfast rarely or never (see Table 2). Notably, while the highest proportion of the children scored in the interquartile-category on the three temperament scales (42.7%–48.1%), there was a large proportion (36.3%) scoring on the “above 75th percentile”-category on the sociable temperament scale. Most households had more than one child, which is consistent with official birth rates. In nearly half of the parents, both had college education or higher, and less than 3% of the mothers were single. Nearly 70% of the mothers were of normal weight, and only about 9% were obese (Table 2). Analyses revealed that child weight and breakfast habits were not significantly associated (*x^2^* = 0.06, *p* = 0.970).

### 3.2. Associations of Temperament with Weight and Breakfast Habits

In Table 3, the temperament categories were associated with the two weight categories of overweight/obesity *vs.* normal weight/underweight (reference). There were overall (unadjusted) significant associations of the externalizing (*p* = 0.025) and sociability temperament categories with overweight (*p* = 0.026), but no significant associations of the internalizing categories (*p* = 0.064). Externalizing temperament (above the 75th percentile) raised the odds for the child to be overweight by 1.16 (unadjusted) and by 1.22 when adjusted for all confounders (Table 3). The slight increase in the OR when adjusting may suggest a suppressor effect. Internalizing temperament (above 75th percentile) lowered the odds for overweight by 0.86 (unadjusted) and 0.82 when fully adjusted. The interquartile-categories of externalizing and internalizing temperament were not significantly associated with child overweight. Interestingly, sociability was significantly associated with child overweight in the interquartile category, raising the odds of being overweight by 1.18 unadjusted and 1.16 when adjusted (Table 3), while the highest category of sociability, above the 75th percentile, was not associated with overweight.

When testing the association of each temperament scale with the frequency of eating breakfast, we found overall (unadjusted) significant associations for the externalizing (*p* = 0.000) and the internalizing (*p* = 0.005) categories. There were no significant associations of sociability with breakfast eating (*p* = 0.368). Externalizing temperament (above the 75th percentile) nearly doubled the (unadjusted) odds for eating breakfast 0 to 3 times per week and raised the odds by 1.65 for eating breakfast only 4 to 6 times per week, compared to eating breakfast every day. Adjusted for all confounders, externalizing temperament raised the odds by 1.57 for eating breakfast 0 to 3 times per week and raised them by 1.43 for eating breakfast 4 to 6 times a week (above 75th percentile). Internalizing temperament (above 75th percentile) raised the odds (unadjusted) for eating breakfast 4–6 times a week by 1.47, but did not change the odds for eating breakfast 0 to 3 times a week. The association of internalizing temperament with eating breakfast was not significant any longer after adjusting for confounders. Internalizing temperament was, however, significantly associated with breakfast eating in the interquartile-category unadjusted, and raised the odds by 1.43 for 0–3 times a week and by 1.26 for 4–6 times a week (Table 4).

**Table 3:** Associations of Child Temperament with Child Overweight/Obesity *versus* Normal weight/Underweight.

Child Overweight or Obesity							
		Unadjusted			Adjusted ^†^		
Temperament Categories		OR	95% CI		OR	95% CI	
Externalizing	75%–100%	1.16 *	1.03	1.31	1.22 **	1.07	1.39
	25%–75%	1.02	0.91	1.13	1.04	0.93	1.16
	0%–25%	-	-	-	-	-	-
Internalizing	75%–100%	0.86 *	0.76	0.98	0.82 **	0.71	0.94
	25%–75%	0.95	0.85	1.06	0.92	0.83	1.03
	0%–25%	-	-	-	-	-	-
Sociability	75%–100%	1.09	0.96	1.24	1.05	0.93	1.20
	25%–75%	1.18 **	1.04	1.33	1.16 *	1.03	1.31
	0%–25%	-	-	-	-	-	-

Note: * *p* ≤ 0.05; ** *p* ≤ 0.01; OR = odds ratio; CI = confidence intervals. Unadjusted: the three temperament scales are not adjusted for each other; ^†^ Adjusted: child gender, number of children in household, maternal BMI, parental education, marital status, and maternal distress (SCL-8).

**Table 4:** Associations of child temperament with frequency of breakfast eating.

		Breakfast 0–3 Times per Week						Breakfast 4–6 Times per Week					
		Unadjusted			Adjusted ^†^			Unadjusted			Adjusted ^†^		
Temperament Categories		OR	95% CI		OR	95% CI		OR	95% CI		OR	95% CI	
Externalizing	75%–100%	1.76 **	1.26	2.47	1.57 *	1.09	2.26	1.65 **	1.31	2.07	1.43 **	1.12	1.83
	25%–75%	1.13	0.81	1.57	1.06	0.75	1.48	1.16	0.93	1.45	1.08	0.86	1.35
	0%–25%	-	-	-	-	-	-	-	-	-	-	-	-
Internalizing	75%–100%	1.43	0.98	2.07	1.07	0.71	1.61	1.47 **	1.16	1.88	1.15	0.88	1.50
	25%–75%	1.43 *	1.02	1.99	1.27	0.90	1.79	1.26 *	1.01	1.57	1.14	0.91	1.43
	0%–25%	-	-	-	-	-	-	-	-	-	-	-	-
Sociability	75%–100%	0.99	0.69	1.43	0.97	0.67	1.41	0.86	0.69	1.09	0.87	0.69	1.10
	25%–75%	1.23	0.87	1.74	1.24	0.87	1.75	0.90	0.72	1.12	0.92	0.73	1.15
	0%–25%	-	-	-	-	-	-	-	-	-	-	-	-

Note: * *p* ≤ 0.05; ** *p* ≤ 0.01; OR = odds ratio; CI = confidence intervals. Unadjusted: the three temperament scales are not adjusted for each other; ^†^: child gender, number of children in household, parental education, marital status, maternal BMI, and maternal distress (SCL-8); Reference category: “Breakfast every day”.

## 4. Discussion

The results from our study showed that child temperament at age five years was slightly associated with concurrent child overweight and moderately so with child breakfast habits. Children high in externalizing temperament had higher odds of being overweight as well as higher odds of not eating breakfast daily. Children high in internalizing temperament were not more prone to being overweight, but had higher odds of not eating breakfast daily. Children with average scores of sociability were more prone to being overweight, while children in the highest or lowest quartiles were not. Children high in sociability did not eat breakfast more or less frequently than children with low scores in sociability.

This study adds important information regarding temperamental risk factors for overweight in the age group of five-year-olds in the general population, particularly with respect to externalizing temperament. Previous studies in this age group found no associations between temperament/behavior and overweight. For instance, a larger population study in four to five-year-old Australian children did not find differences in mental health/behavior between overweight/obese and normal weight children [49]. In a similar vein, studies including children between three to eight years [46] and children between two and five years did not find associations between temperament dimensions (shyness, sociability, emotionality, rhythmicity, irritability, activity) and BMI Z-scores [44]. A longitudinal study that measured temperamental dimensions similar to ours (activity, persistence, soothability, smiling, and fear) in infancy and weight gain and adiposity at six to eight years did not result in significant associations either [51]. Our findings regarding average scores of sociability being associated with overweight are broadly in line with earlier findings where surgency/extraversion was associated with greater weight gain and overweight [33,36,38]. They are also compatible with a model of non-linear associations where those scoring the highest on sociability may have lower weight because of their higher activity. A strength of our study compared to previous ones is the greater power to detect even small associations.

There are several mechanisms that may explain the relation of externalizing temperament with overweight. One of the most important traits underlying externalizing temperament is (lack of) self-regulation, the child’s ability to regulate his or her own emotions and behavior. This ability is present from early infancy and evolves further as the child develops [23]. Lack of self-regulation in children has important repercussions; such children have problems calming themselves when upset, and entertaining themselves when bored. Indeed, a recent article showed that attention and perseverance, both aspects of self-regulation, were weaker in overweight children [62]. These temperament traits increases the risk that the children fall back on readily available comfort through palatable foods offering quick satiation, such as sweets and snacks, which in turn are obesogenic [37,43].

Another mechanism proposed to explain the relation between child temperament and overweight is parental use of food to soothe and calm the externalizing, temper-tantrums throwing child [55]. This explanation is supported by our previous finding, where emotional, externalizing temperament was associated with mothers beginning to bottle-feed the externalizing child earlier, introduce solid foods earlier, and letting the child consume sweet foods and drinks more frequently [40,51,52]. Not only can feeding in order to soothe lead to increased calorie intake, but it can also disrupt the child’s self-regulation of food intake whereby external food cues become more important than internal food cues [36,41,52,63,64].

With respect to explaining the association of externalizing temperament with breakfast habits, we cannot find comparable studies. This topic has hitherto only been investigated in youths and adolescents [65,66,67]. Regarding mechanisms, we are bound to speculate based on the existing literature [68]. Five-year olds depend on the adults in their environment to prepare and serve food and to offer regular family meals. We do not know whether the children eating breakfast fewer times than daily were offered breakfast or not, but we controlled for known risk factors for inadequate child feeding such as parental education, maternal marital status and number of children in the household. At the same time, children can regulate their food intake by refusing to eat—which is consistent with the defiance and acting-out behavior captured by externalizing temperament. Indeed, some studies found that children with externalizing temperament were choosy eaters, and struggled with the mother for control over food intake [69]. Refusal to eat breakfast could also be a symptom of an evolving eating disorder [70].

As in all studies, there are limitations that should be addressed. The design was cross-sectional, hence, we do not know whether temperament influenced weight and breakfast regularity or whether weight and breakfast regularity influenced temperament. For example, it is known that overweight children are teased by others and can become more depressed. In addition, not eating a proper breakfast can lead to depressed mood and attention problems. Hence, there is a probably a mutual association [45]. Another concern is that all information was mother-reported, introducing possible respondent-bias such as social desirability. In particular, mother-reporting of child weight can be biased. Because of low prevalence, we could not differentiate between overweight and obesity. Another limitation is that the entire MoBa study sample comprises more highly educated, older, married or cohabitating, non-minority, non-smoking women compared to all the mothers in Norway giving birth during the same decade [71,72]. In spite of this bias, the associations of important exposures with adverse pregnancy outcomes in the MoBa study are the same as in the entire birthing population of Norway [71,72]. Moreover, the frequency of overweight and obesity in our study is broadly similar to that found in other Norwegian studies [73,74]. The temperament dimensions only explained a small share of the variability of weight status and breakfast habits. At the same time, in view of the positive results and the sample size, our study adds important information to the existing body of literature.

Parents and teachers should be sensitized to the finding that children showing externalizing or acting-out temperament may be at risk for problematic eating habits and overweight. Although introducing a systematic screening of temperament, weight and eating habits would be difficult to introduce in many countries, children with externalizing temperament are easy to spot and school-health staff may explore these children’s eating habits before they become overweight. Screening is particularly important given the finding that treatment of childhood obesity is difficult and prevention must therefore be prioritized.

## 5. Conclusions

Our study once again reveals the importance of externalizing temperament for overweight and eating habits in children. Because children’s weight development undergoes the adiposity rebound between age five and age seven years, future studies, including the MoBa study, ought to explore temperamental predictors of weight status and change during this period. It is particularly important to uncover the mechanisms relating externalizing temperament to weight and food intake. In addition to merely behavioral measures of food consumption, issues related to food and nutrient preferences, appetite, sensitivity to reward, eating styles, eating disorders, as well as satisfaction obtained by eating ought to be explored.
